# Caddisfly Larvae are a Driver of Plastic Litter Breakdown and Microplastic Formation in Freshwater Environments

**DOI:** 10.1002/etc.5496

**Published:** 2022-11-18

**Authors:** Katey Valentine, Richard Cross, Ruairidh Cox, Gina Woodmancy, Alistair B. A. Boxall

**Affiliations:** ^1^ Department of Environment and Geography University of York Heslington UK; ^2^ UK Centre for Ecology and Hydrology Wallingford UK

**Keywords:** Microplastics, aquatic invertebrates, benthic macroinvertebrates, environmental fate, freshwater, caddisfly, plastic

## Abstract

Plastic litter is now pervasive in the aquatic environment. Several marine and terrestrial organisms can fragment plastic with their feeding appendages, facilitating its breakdown and generating microplastics. However, similar studies with freshwater organisms are extremely limited. We explored the interactions between the caddisfly larvae *Agrypnia* sp. and polylactic acid (PLA) film. The use of plastic by larvae to build their protective cases was investigated, along with their ability to fragment the plastic film as they do with leaf litter. Caddisfly consistently incorporated PLA into their cases alongside leaf material. They also used their feeding appendages to rapidly fragment PLA—forming hundreds of submillimeter‐sized microplastics. Although larvae showed a preference for leaf material when constructing cases, plastic use and fragmentation still occurred when leaf material was replete, indicating that this behavior is likely to occur in natural environments that are polluted with plastics. This is thought to be the first documented evidence of active plastic modification by a freshwater invertebrate and therefore reveals a previously unidentified mechanism of plastic fragmentation and microplastic formation in freshwater. Further work is now needed to determine the extent of this behavior across freshwater taxa and the potential implications for the wider ecosystem. *Environ Toxicol Chem* 2022;41:3058–3069. © 2022 The Authors. *Environmental Toxicology and Chemistry* published by Wiley Periodicals LLC on behalf of SETAC.

## INTRODUCTION

In 2020, approximately 367 million tons of plastic were produced globally, and production is expected to continually increase (Plastics‐Europe, 2021). Mismanagement of this plastic during its end‐of‐life stage is predicted to result in 156–266 million MT yr^–1^ of mismanaged plastic municipal waste being leaked to the environment by 2026 (Lebreton & Andrady, 2019; Zhang et al., 2021). Types of plastic litter found in rivers and other freshwater systems can range from large items, such as whole bottles and carrier bags, to smaller meso‐ and microsize particles (Gallitelli & Scalici, 2022; Liro et al., 2020). Most of the microplastics in freshwater systems are secondary microplastics—resulting from the gradual degradation and fragmentation of larger plastic items in the environment (Vaid et al., 2021; Wong et al., 2020; Yang et al., 2021). The formation of secondary microplastics by abiotic factors, such as mechanical stress, photodegradation, thermal stress, and degradation of chemicals, along with biodegradation by microorganisms living on plastic surfaces, is well documented (Hossain et al., 2019; Shah et al., 2008; Zettler et al., 2013; Zhang et al., 2021).

An emerging but poorly understood mechanism of plastic fragmentation is its breakdown by larger organisms such as invertebrates, which can alter plastics through biting, chewing, and digesting (So et al., 2022; Zhang et al., 2021). Referred to as biofragmentation, this process has been observed in marine systems. For example, the marine amphipod *Orchestia gammaerellus* and the sea urchin *Paracentrotus lividus* can degrade mesoplastic materials into smaller fragments using their feeding appendages (Hodgson et al., 2018; Porter et al., 2019). Marine polychaetes and isopods can form plastic fragments from their burrowing behavior into expanded polystyrene (Davidson, 2012; Jang et al., 2018), and evident bite marks in plastic litter washed up on beaches are commonly seen (Carson, 2013). Biofragmentation can also occur internally within an organism's digestive system and is predominantly seen in aquatic crustaceans. For example, the amphipod *Gammarus dubeni*, Antarctic krill (*Euphausia superba*), Atlantic ditch shrimp (*Palaemon varians*), lobster (*Nephrops norvegicus*), and crab (*Carcinus maenas*) can internally degrade larger plastics into smaller particles, including micro‐ and nanoplastics (Cau et al., 2020; Mateos‐Cárdenas et al., 2020; Murray & Cowie, 2011; Saborowski et al., 2019; Torn, 2020; Watts et al., 2015). Small amounts of internal plastic fiber fragmentation have also been documented in larvae of the freshwater dragonfly *Anax imperator* (Immerschitt & Martens, 2020). Some terrestrial insects have recently gained attention for their ability to rapidly degrade plastic films and foams, resulting in microplastic formation, and have been investigated for their bioremediation potential (see, Billen et al., 2020; Bombelli et al., 2017; Brandon et al., 2018; Büks et al., 2020; Helmberger et al., 2022; Peng et al., 2022; Wang et al., 2020). Given the taxonomically broad range of organisms known to fragment plastic, and the fact that many plastics become deposited in the benthic environment, (Egessa et al., 2020) this mechanism of microplastic formation may be an important contributor to microplastic generation in the environment. However, research into the biofragmentation of plastic is in its infancy, and studies addressing this process in freshwater invertebrates are particularly lacking (So et al., 2022), even though freshwater rivers are one of the largest contributors of microplastics to marine systems (González‐Fernández et al., 2021; Lebreton et al., 2017).

Recently, interactions between plastic litter and the larvae of caddisfly (order Trichoptera) have been documented. Many caddisfly larvae build portable cases from hard particles such as mineral grains, or organic material like leaves and other plant material, which they attach together using self‐produced silk (Holzenthal et al., 2015). Microplastics were found in the hard mineral‐grain cases of caddisfly from several rivers (Alvarez Troncoso et al., 2022; Ehlers et al., 2019; Gallitelli et al., 2020, 2021; Tibbetts et al., 2018), and the active incorporation of hard plastic particles into cases has since been confirmed in laboratory studies with three different caddisfly species (Ajiboye, 2021; Ehlers et al., 2020; Gallitelli et al., 2021). Many caddisfly larvae build with flexible plant material, such as leaves, which they often first fragment into the desired size and shape using highly sclerotized mandibles (Wiggins, 1960). To date, all comprehensive studies have looked only at the interactions of caddisfly with hard plastic particles; they have also mainly focused on caddisfly species that build from hard mineral grains and have never assessed the ability of caddisfly to use or fragment softer plastic films. This ability of some caddisfly to fragment material, along with the documented interactions of caddisfly with plastic litter and the discovery of the rapid degradation of plastics by terrestrial insect larvae, raises the questions “do caddisfly larvae utilize and fragment the flexible plastic film they encounter in the environment?” and as a result, “are they a potential important contributor to microplastic formation in freshwater systems?”

Therefore, we explored the interactions between the freshwater caddisfly larvae *Agrypnia* sp. and polylactic acid (PLA) film. *Agrypnia* sp. is common in still and slow‐running freshwater systems throughout Europe and North America (GBIF‐Backbone‐Taxonomy, 2021) and builds its case from pieces of fragmented flexible organic material (Supporting Information, Figure S1). Polylactic acid is a bio‐based polyester traditionally marketed as a biodegradable alternative to conventional plastic—which has led to its increased use in food packaging and agricultural mulching films as an “eco‐friendly” alternative (Akhir & Mustapha, 2022; Ncube et al., 2020). However, even though PLA is biodegradable in industrial compositing systems, in aquatic environments such degradation is reported to be extremely slow and therefore thought to pose pollution risks similar to those of conventional plastics (Ncube et al., 2020). The aim of the present study was to establish whether the benthic larval invertebrate *Agrypnia* sp. would actively interact with PLA film to build a protective case, and if so, whether these interactions would lead to the fragmentation of plastic film and the formation of microplastic particles.

## MATERIALS AND METHODS

Throughout our study, plastic particles with a maximum Feret diameter between 1 and 10 mm are defined as mesoplastics and those between 1 and 1000 µm are defined as microplastics, as recommended by Hartmann et al. (2019).

### Test organisms and materials

*Agrypnia* sp. larvae were obtained from the online retailer Blades Biological, and their identity was confirmed using a taxonomic key (Wallace et al., 2003). Experimental work with caddisfly larvae was approved by the Environment and Geography Department Ethical Review Committee at the University of York (Heslington, UK). After arrival, organisms were maintained at 15 °C under a 12:12‐h diurnal light:dark cycle in a glass aquaria containing approximately 10 L of artificial pond water (Naylor et al., 1989) and fed ad libitum with algal pellets comprising nutrient agar, cellulose, and *Chlorella* powder (the full recipe is given in the Supporting Information, Methods S1; Kampfraath et al., 2012). The plastic film used was white, commercially available, food‐contact‐grade PLA bags 45 µm in thickness, and the polymer identity was confirmed using a Nicolet iS10 spectrometer (Supporting Information, Figure S2). The PLA film had a density of 1133 kg/m^3^ and was therefore negatively buoyant. Previous studies report higher levels of interaction between invertebrates and plastic when the plastic is first microbially conditioned (Hodgson et al., 2018; Porter et al., 2019). Prior to use in our study, the PLA was therefore conditioned for 3 weeks by placing material in 240‐ × 240‐ × 130‐mm custom‐built stainless‐steel cages (mesh aperture 0.57 mm) below the surface of the River Ouse, UK (54°00′30.7″N, 1°11′28.7″W) in July 2020. After 3 weeks, the cages were removed from the river, and the plastic was rinsed with Milli‐Q water and stored at −80 °C until use.

Oak (*Quercus robur*) leaves were used as a representative natural organic building material. Leaves were sun‐dried green leaves from an organic woodland (Hanging Wood, Highfields/Woodlands, Yorkshire, UK); they were obtained online and were soaked in Milli‐Q water for 2 days at 4 °C to rehydrate them. Previous efforts by the authors to form a biofilm on the surface of leaf material with the same methods used for plastic was found to substantially alter the structural integrity of the material, making it possible but highly challenging to handle and perform surface area measurements. For this reason, to ensure accurate measurements could be performed, in situ microbial conditioning of leaf material was not carried out in the present study. Although some biofilm formation may have occurred on leaf material during the experimental exposure period (Artigas et al., 2011), some studies have found that very little growth of bacterial, fungal, and yeast colonies occurs on *Q. robur* leaves before approximately 8 days of submersion in natural water (Sampaio et al., 2001). The experimental design of the present study therefore represents a choice between plastic film already present in the environment and leaf litter that has recently entered the environment after falling from surrounding trees. For experimental use, plastic and leaf material were cut into 6‐ × 3.4‐mm pieces. The microbially colonized PLA weighed 51.2 ± 0.91 µg/mm^2^, and the rehydrated leaf material weighed 175.5 ± 20.8 µg/mm^2^.

### Experimental design

Preliminary trials showed that larvae used approximately 26 (6‐ × 3.4‐mm) pieces of material (leaf or plastic) to build a new case, and that case building was most successful when leaf material was also available. Our study therefore consisted of two treatment groups: a “material‐limited” group whereby larvae were given 13 PLA and 13 leaf pieces—to determine whether larvae would use PLA if doing so was the only way to build a completely new case; and a “material‐replete” group where larvae were given 26 PLA and 26 leaf pieces—to determine whether they would use PLA when there was sufficient organic building material available for case construction. Each treatment group contained 12 replicates. Twelve control replicates/treatment group, which consisted of an identical setup without the larvae, were also run in parallel. Before being placed into experimental jars, larvae were gently removed from their original cases using a blunt glass pipette. Larvae were randomly assigned to treatments so that there was no significant difference in the original case length of larvae between the material‐limited and material‐replete treatments (*t* = 1.03, *df* = 22, *p* = 0.31). The experimental treatments ran for 6 days with larvae placed in glass jars containing 150 ml artificial pond water, building material, and a 0.95‐g algae pellet, and were maintained under the same light and temperature conditions used for organism acclimation. During this time, all jars were covered with aluminum foil and continuously aerated using an airline and hypodermic needle. To maintain high water quality, a 90% water change was made after 2 and 4 days, and water from these changes was stored. After 6 days, larvae were removed from their new cases and euthanized by storing at −80 °C. Newly built cases were stored at −20 °C along with any mesoplastic and leaf material remaining in the jar, which was recoverable by hand using steel forceps. The remaining water was collected, added to that from the water changes, and stored in a Duran bottle at 4 °C until analysis.

### Material analysis

Cases were measured, photographed, and deconstructed to separate out plastic and leaf pieces. Plastic and leaf pieces from the cases and those recovered by hand from the jar were then imaged using an Epson ET‐2720 scanner, and their surface area was quantified using Image J Ver 1.53a using a thresholding technique. These pieces were compared visually with control pieces to determine whether they were intact or whether they showed evidence of chewing or fragmentation.

To determine whether micro‐PLA fragments had been formed, all exposure water samples were made up to 800 ml volume with Milli‐Q water (filtered to 0.2 µm). To minimize microplastic adhesion to each other and to the bottle sides, 1 ml of a 10% (v/v) TWEEN®20 solution in Milli‐Q water was then added to each bottle. Each sample was shaken before approximately 50% of the sample was passed through black Cyclopore 0.2‐µm polycarbonate filters using a glass vacuum filtration pump. The exact volume of water filtered was determined by weighing water samples before and after samples were decanted for filtration. The filter was then imaged using a Zeiss Axio Zoom V16 microscope coupled with an Axio Zoom 105 color camera and Zen (Ver 2.0) software. Images were processed in Image J, with white micro‐PLA particles identified visually within each image and measured using a thresholding‐based technique for each individual particle. The number of counted micro‐PLA particles was subsequently scaled to the full sample exposure water volume.

### Quality control and assurance

Throughout our study, external microplastic contamination was minimized wherever possible by rinsing all apparatus and glassware three times with 0.2‐µm‐filtered Milli‐Q water and keeping all samples and apparatus closed to the air or covered in aluminum foil. Further quality control steps were taken to ensure that the micro‐PLA count data were accurate. First, micro‐Fourier transform infrared spectroscopy (μFTIR) analysis of two treatment samples was performed and confirmed that the visually observed white microparticles were PLA—details of the µFTIR methodology are given in the Supporting Information, Methods S2. Analysis of one control sample using µFTIR found that no PLA particles were present. Second, to further account for any white particles that still may have been misidentified as PLA, exposure water of control samples was filtered and visually analyzed in the same way as treatment samples. In the exposure water of control replicates, the average scaled number of particles that would have been visually identified as PLA was 0.3 in material‐limited controls (*n* = 10) and 1.2 in material‐replete controls (*n* = 11). This value was deducted from the scaled number of particles counted in each treatment replicate.

### Statistical analysis

Statistical analysis of data and figure construction was performed in R Studio Ver 1.2.1335 with packages ggplot2, car, and FSA. Data were analyzed to compare the amount of plastic and leaf used within and between treatments (paired and unpaired *t*‐tests), along with the proportion and size of intact and chewed meso‐PLA pieces (unpaired *t*‐test and Kruskall–Wallis test), and the number and size of micro‐PLA particles created (unpaired *t‐*test and Mann–Whitney *U* test). Relationships between the number of micro‐PLA particles and the remaining surface area of meso‐PLA pieces were also investigated with linear regression analysis. All data residuals were tested for normality using a Shapiro–Wilk test and for equal variance with either a Levene or a Breusch–Pagan test. When data did not meet these assumptions, either they were transformed or else a nonparametric test was carried out if assumptions were still not met after transformation. Details of the tests and transformations carried out for each of the endpoints are outlined in the Supporting Information, Table S1. The significance level was set at 0.05.

## RESULTS

### Incorporation of plastic film into cases

**Figure 1:**
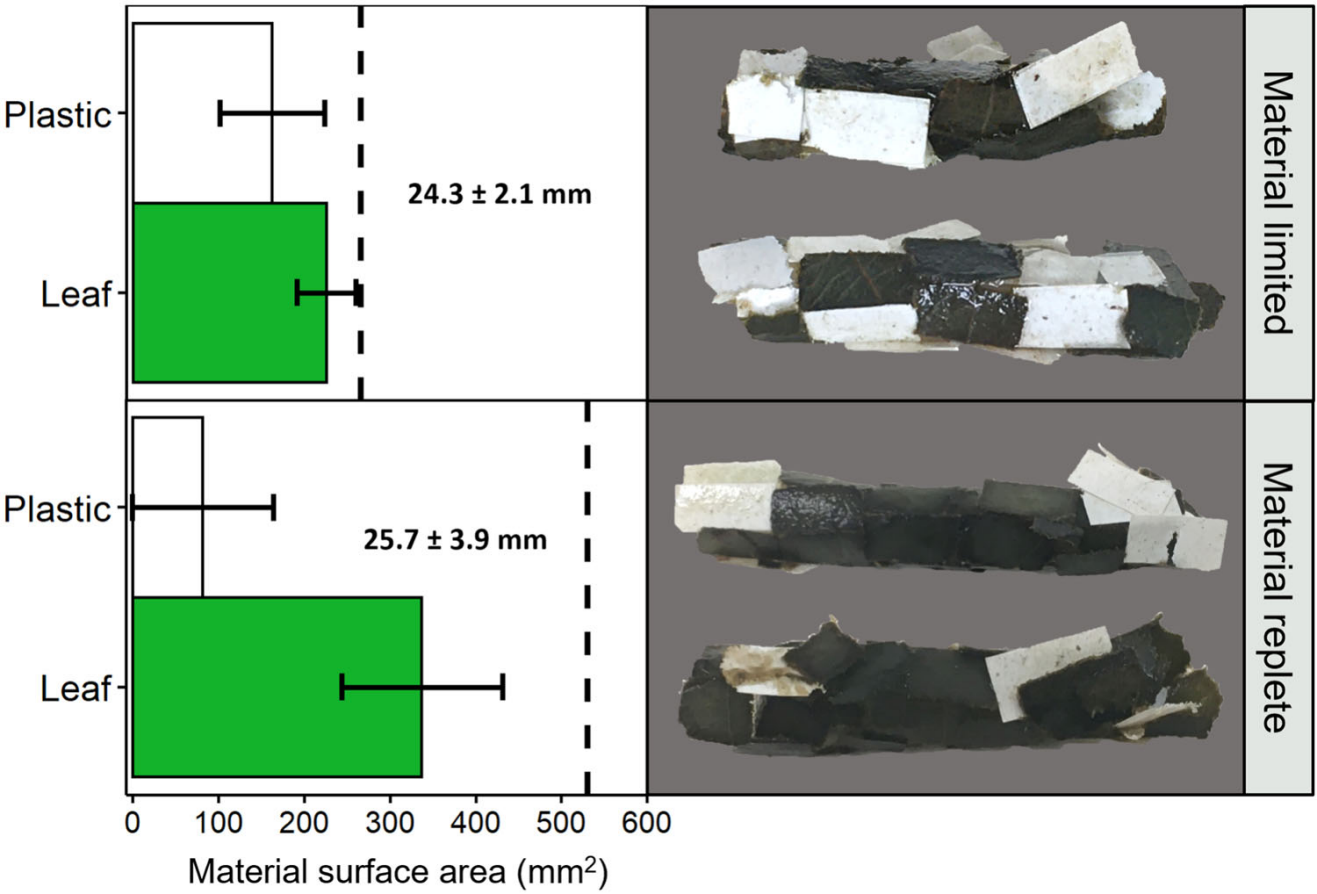
(Left) Average amount of leaf and polylactic acid (PLA) material used by caddisfly larvae to construct new cases for material limited and material replete treatments. Error bars show standard deviation. Text on each plot is the average length ± SD of the newly built cases. Dashed line on each plot indicates the total amount of plastic and the total amount of leaf available to the larvae in each treatment. For material limited treatment (*n* = 11), for material replete (*n* = 12). (Right) Examples of newly constructed cases made from leaf and plastic material by larvae in each treatment group; dark green parts are oak leaf and white parts are PLA film.

After 6 days all larvae had used the provided material to construct a new case (Figure 1). With the exception of three larvae in the material‐limited and one in the material‐replete treatment, which built their case against the side of the glass jar, all larvae built complete portable cases. There was no significant difference in the length of new cases (*U* = 55.5, *p* = 0.35) or total surface area of the material (leaf + plastic; *t* = −0.68, *df* = 21, *p* = 0.51) used to build them across treatments. There was also no significant difference in the wet weight of larvae between treatments at the end of our study (*t* = −0.04, *df* = 22, *p* = 0.97). All 12 larvae in the material‐limited treatment and 11 of the 12 larvae in the material‐replete treatment incorporated PLA into their new case. Overall, larvae tended to favor leaf material over plastic. This preference was present when natural material was limited (cases on average contained 41% plastic and 59% leaf) and became clearer when natural material was replete (cases on average contained 17% plastic and 83% leaf); in both treatments, cases consisted of significantly more leaf than PLA (*t*_(ML,MR)_ = 3.36, 7.65, *df*_(ML, MR)_ = 10, 11, *p*_*(*ML&MR)_ = 0.01, 9.93E^–6^); ML represents material limited and MR represents material replete. The amount of each material available that was used by larvae differed significantly across treatments, with larvae in the material‐limited treatment incorporating significantly more PLA into their new cases compared with larvae in the material‐replete treatment (*t* = 2.64, *df* = 21, *p* = 0.02). In contrast, significantly more leaf was used to build cases by larvae in the material‐replete treatment compared with the material‐limited treatment (*t* = −3.84, *df* = 14.14, *p* = 1.76E^–3^). Of particular note was that of the 11 larvae that used PLA in the material‐replete treatment, 9 of them still had unused pieces of leaf material remaining loose in the exposure jar at the end of our study.

### Plastic fragmentation and microplastic formation

**Figure 2:**
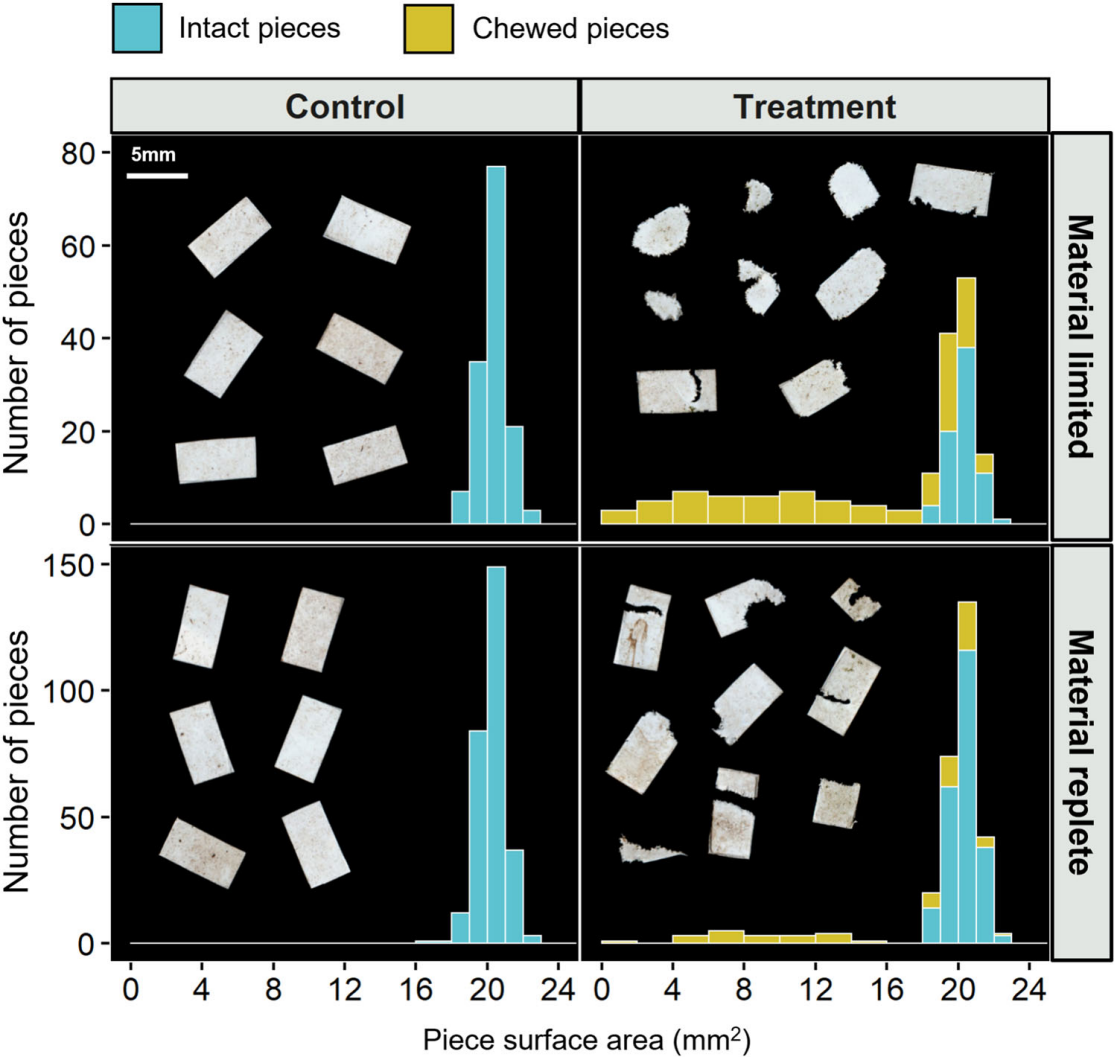
Size distribution of meso‐polylactic acid (PLA) pieces (>1 mm) recovered from caddisfly cases and exposure water in material limited and material replete treatments, displayed as a stacked histogram. Color coding denotes the number of pieces within each bin that were either intact or had visual evidence that chewing had occurred; *n* = 11 for both treatment and control groups. Note the different scale for limited and replete treatments. Inlets into control plots are example images of intact meso‐PLA pieces, and inlets into treatment plots are examples of chewed pieces from each treatment group. Scale bar = 5 mm for all PLA piece images.

Chewing and fragmentation of PLA film by *Agrypnia* sp. larvae was clearly evident. In both treatments many of the meso‐PLA pieces incorporated into cases, as well as those remaining in the exposure jar, showed clear signs of chewing, which was not observed in pieces from control treatments (Figure 2). In the material‐limited treatment 52% ± 31% (mean ± SD) of the provided meso‐PLA pieces remained intact, with no visual signs of chewing. This was significantly less (*t* = −2.87, *df* = 20, *p* = 0.01) than in the material‐replete treatment, in which 85% ± 14% remained intact. The extent of chewing on PLA pieces was highly variable, with some pieces extensively chewed and broken into small fragments and others only slightly chewed around the edges. Across all treatment and control groups the size (mean ± SD) of intact meso‐PLA pieces was 20.3 ± 0.8 mm^2^; chewed pieces in the material‐limited treatment were 14.3 ± 6.5 mm^2^ and 16.4 ± 5.7 mm^2^ in the material‐replete treatment. A significant difference was seen between the size of intact, material‐limited‐chewed and material‐replete‐chewed PLA pieces (*H* = 138.76, *p* = 2.2E^–16^), and a post hoc analysis (Dunn's test) revealed differences among all three of these groups (*p* < 0.01 for all). Further evidence for the plastic‐fragmentation behavior of *Agrypnia* sp. larvae, and the use of their mandibles for this, was seen outside of the main study when a larva involved in preliminary investigations was captured in a video chewing and fragmenting the PLA film before incorporating the material into its case (Supporting Information, Video S1).

**Figure 3:**
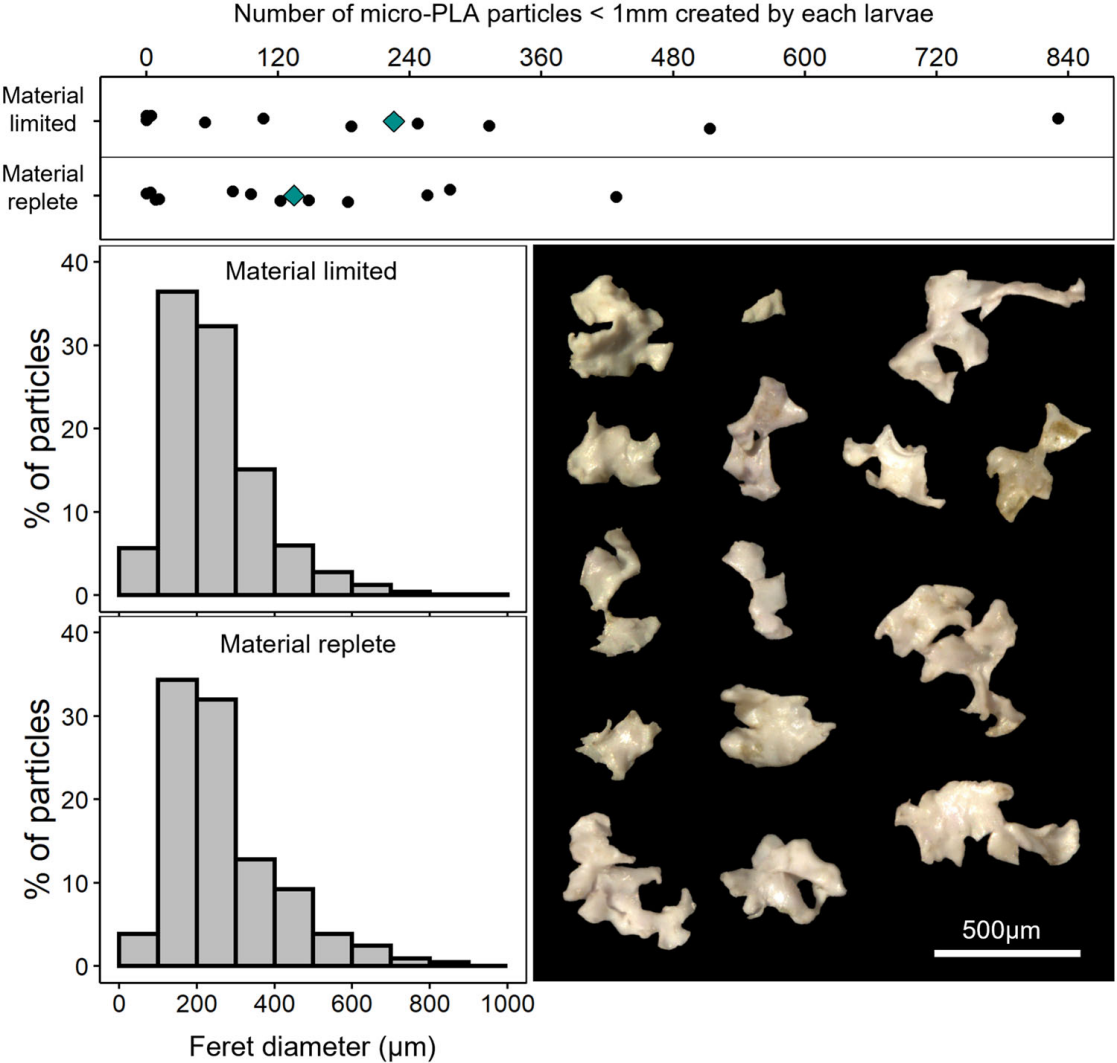
(Top) Scaled number of polylactic acid (PLA) microplastic fragments (<1 mm) formed by larvae over the 6‐day study. Black dots show the number of PLA microparticles for each replicate in either material‐limited (*n* = 10) or material‐replete (*n* = 12) treatment. Blue diamonds show the average for each treatment. (Left) normalized size distribution of all microplastic fragments found in the exposure water of replicates from either material‐limited (*n* = 1440) or material‐replete (*n* = 1054) treatments. (Right) example images of microplastic fragments from both treatments.

Analysis of exposure water revealed that the fragmentation of PLA film by *Agrypnia* sp. larvae led to the formation of micro‐PLA particles (less than 1 mm in diameter). The number of micro‐PLA particles found in the filtered exposure water varied considerably among replicates in both treatments (Figure 3). Larvae in the material‐limited treatment formed 225 ± 269 (mean ± SD) micro‐PLA particles throughout the study, which did not statistically differ from the 134 ± 133 particles formed by larvae in the material‐replete treatment (*t* = 0.58, *df* = 20, *p* = 0.57). As would be expected, the total surface area of meso‐PLA pieces recovered from the case and exposure jar of each replicate was a significant predictor for the number of micro‐PLA particles found for both the material‐limited (*R* ^2^ = 0.87, *F*_(1,7)_ = 46.93, *p* = 2.42E^–4^) and material‐replete (*R* ^2^ = 0.63, *F*_(1,9)_ = 15.04, *p* = 3.74E^–3^) treatments, with a higher number of micro‐PLA particles found in replicates from which a lower amount of meso‐PLA surface area was recovered (Supporting Information, Figure S3). Although larvae size did not differ significantly between the two treatments, small natural variation in their size across all individuals was present. Interestingly, however, the size of the caddisfly was not a significant predictor for the number of microplastics formed in either of the treatments, material limited (*R* ^2^ = 0.24, *F*_(1,8)_ = 2.47, *p* = 0.15), or material replete (*R* ^2^ = 0.22, *F*_(1,9)_ = 2.53, *p* = 0.15).

The size (maximum ferret diameter) of visually identified micro‐PLA particles formed by larvae ranged between 35.61 and 927.86 µm. FTIR analysis to a resolution of 6.25 µm did not identify any PLA microparticles below this size range; however, it should be noted that although unlikely, it is possible that micro‐PLA particles that fell below 6.25 µm could have been generated. For both treatments, the most common size of micro‐PLA particle was 100–300 µm (Figure 3), with 68.7% and 66.3% of particles measuring between 100 and 300 µm for material‐limited and material‐replete treatments. The average particle size (mean ± SD) for material‐limited (244.1 ± 121.2 µm) and material‐replete (228.6 ± 142.1 µm) treatments was very similar. Even so, a significant statistical difference in particle size across treatments was detected (*U* = 697142, *p* = 5.66E^–4^). However, interpretation of the size‐frequency distribution and the descriptive statistics suggests that the large number of particles used for the analysis may have led to high power and sensitivity of the statistical analysis, which exaggerated small differences that are unlikely to be biologically meaningful.

## DISCUSSION

The biofragmentation of plastic by larger organisms such as macroinvertebrates is beginning to be recognized as a potentially important pathway for plastic litter breakdown and microplastic formation in the environment (So et al., 2022). However, data on the occurrence of biofragmentation in freshwater systems are severely limited. To our knowledge, our study is the first to document the active fragmentation of plastic films by a benthic freshwater invertebrate—which led to the formation of hundreds of microplastic particles. Throughout the present study *Agrypnia* sp. larvae consistently interacted with flexible PLA film, and, along with leaf material, they used their mandibles (shown in the Supporting Information, Video S1) to fragment the plastic and used it to build a new protective case.

### Larvae interaction with and use of plastic films

The use of plastic to build protective cases is in line with previous studies with other caddisfly species, which found that larvae incorporated both natural and plastic particles into their cases when a mixture was available (Ajiboye, 2021; Ehlers et al., 2020; Gallitelli et al., 2021). In the present study *Agrypnia* sp. larvae generally showed a preference for leaf as a building material; however, their use of PLA occurred even when leaf material was replete, and many larvae that had incorporated PLA into their case still had unused leaf material remaining. Although it may be that the larvae's use of plastic was random, with larvae including material as they encountered it, PLA may have possessed both desirable and undesirable properties as a building material—resulting in a trade‐off between the benefits and drawbacks if its use. For example, a desirable property of PLA could have been its apparent smoothness compared with leaf material (Supporting Information, Figure S4); as some caddisfly show a strong preference for smoother materials because they can more easily attach their silk to the surface (Okano & Kikuchi, 2009; Okano et al., 2012). To reduce the chance that their case will be consumed by other shredding organisms, caddisfly are also known to favor material that has a lower palatability and perceived nutritional quality (Moretti et al., 2009; Rincón & Martínez, 2006). Even though the PLA in our study were microbially conditioned for 3 weeks and the leaf material was not, caddisfly do often feed on leaf detritus, including *Quercus* sp., (González & Graça, 2003) and may therefore have still perceived the PLA as less palatable than the leaf. Although the smoothness and lack of palatability may therefore have encouraged the use of plastic in case construction, in contrast, the greater toughness of PLA compared with *Q. robur* leaves (Campanella et al., 2013; Jeong et al., 2018; Mirkhalaf & Fagerström, 2021; Vanstrom, 2012) is likely to be an undesirable property of PLA, because larvae would need to expend more time and energy fragmenting it into the desired shape and size compared with leaf material. After initially interacting with PLA and determining its toughness, this may therefore have been a deterrent to its use. This is supported by the meso‐PLA pieces found loose in the exposure jar—many of which had been partially chewed or fragmented before being abandoned. As discussed, the design of our study represents a choice between plastic film already present in the environment and leaf litter that has recently entered the environment after falling from surrounding trees. Given that the leaf material in our study was not microbially conditioned in the same way as plastic, it should be noted that although the results we present may be applicable for caddisfly interactions with organic and plastic material more generally, further experimental work to test the preferences of caddisfly between plastic and natural material that has been submerged in the environment for longer would be needed to confirm this. Furthermore, other types of leaf from different tree species, as well as PLA film that has been submerged in the environment for longer or shorter periods, could elicit a different response, and additional studies to determine the role of these factors in the behavior of caddisfly would also be useful.

Macro‐ and meso‐sized plastic films are already abundant in freshwater systems (Blettler et al., 2017; Lahens et al., 2018; Winton et al., 2020) and the increasing use of PLA, specifically in applications such as agricultural mulching films (Akhir & Mustapha, 2022), means that its presence in the environment is only likely to grow. Caddisfly have previously been found to be associated with biodegradable plastics in a riparian stream (Artru & Lecerf, 2019), and the presence of microplastics in caddisfly cases collected from the field (Alvarez Troncoso et al., 2022; Ehlers et al., 2019; Gallitelli et al., 2020, 2021; Tibbetts et al., 2018) further confirms that caddisfly do interact with plastic in their natural environment. These previous findings, coupled with the tendency we found of larvae to interact with, use, and fragment plastic even when natural material was abundant therefore indicate that this behavior is likely to occur when caddisfly encounter plastic film in their natural habitat. Furthermore, evidence from our study suggests that even if larvae do not ultimately incorporate plastic into their cases, they may still interact with and “test out” material by beginning to fragment it.

### Potential impacts of caddisfly–plastic interactions

The consequences for *Agrypnia* sp. individuals of interacting with plastic film are unclear, but there is the potential for several favorable and adverse effects. Caddisfly are common prey for larger organisms, and the design of their cases can alter the extent to which they are preyed on (Ferry et al., 2013; Johansson, 1991; Nislow & Molles, 1993). For example, visibility is an important factor in the rate at which they are attacked by fish (Otto & Svensson, 1980), and the inclusion of plastic litter, which is often brightly colored (Lu et al., 2021; Manikanda Bharath et al., 2021), may increase their vulnerability to visual predators. Furthermore, the structural strength of mineral‐grain cases is known to be reduced by the inclusion of microplastics (Ehlers et al., 2020), which can decrease the chance of survival when larvae are attacked (Otto, 1987). Nevertheless, the toughness and lower susceptibility of plastic film to microbial degradation compared with leaf material have the potential to improve the robustness of organic cases and therefore provide increased protection for the larvae. Plastic inclusion in cases has also been shown to increase case buoyancy due to the lower density of plastic compared with mineral grains (Ehlers et al., 2020). In the present study, PLA film was less heavy than leaf material, and its inclusion would therefore have made cases lighter than those constructed only from leaf. Many other plastic films, such as low‐density polyethylene and polypropylene, are less dense than PLA, and their inclusion in larvae cases would likely result in even larger differences in case weight. A lighter case may have both negative and positive consequences for the larvae; whereas heavier cases can provide greater stability in water currents (Delgado & Carbonell, 1997; König & Waringer, 2008), successful prey capture was found to be significantly greater in larvae inhabiting lighter cases (Otto, 1987). Determining plastic ingestion by larvae was beyond the scope of our study, but given the extent of chewing, the large number of microplastics formed by some larvae, and the known ingestion of microplastics by caddisfly larvae (López‐Rojo et al., 2020; Windsor et al., 2019; Winkler et al., 2022), a degree of plastic ingestion would not be unexpected and has the potential to cause considerable adverse effects (López‐Rojo et al., 2020). Furthermore, even if microplastic ingestion did not occur, interactions with harmful chemicals often associated with plastics, such as phthalates, persistent organic pollutants, and toxic metals (Rochman, 2015), could pose an exposure risk to larvae during plastic fragmentation and during their prolonged interaction with the plastic after its incorporation into their cases.

Caddisfly are important members of freshwater ecosystems, with an important role in food webs, as well as carbon and nutrient cycling (Morse et al., 2019). Their fitness is known to substantially influence key ecosystem process such as leaf litter decomposition (López‐Rojo et al., 2020), and changes to larvae health and survival could therefore have implications for the wider ecosystem. Furthermore, in the present study caddisfly larvae transformed plastic debris measuring 6 mm (maximum diameter) into microplastics measuring between 36 and 928 μm (maximum diameter). This biofragmentation process would therefore make plastic litter more bioavailable to benthic organisms only capable of ingesting smaller particles, such as Asellidae isopods and chironomid larvae, which show a higher ingestion of microplastics <50 μm (Pan et al., 2021). Plastic may even become more bioavailable to species such as *Gammarus duebeni*, which are common amphipods in rivers throughout the United Kingdom and are known to internally fragment 10–45‐μm microbeads into even smaller fragments (Mateos‐Cárdenas et al., 2020)—potentially leading to even further plastic breakdown. Microplastic ingestion can lead to a range of negative effects for individual organisms (Guimarães et al., 2021; Karami et al., 2016; Ziajahromi et al., 2018); it can also considerably alter benthic freshwater community structures (Rauchschwalbe et al., 2022). The impacts of this microplastic influx (facilitated by caddisfly larvae) on the surrounding freshwater community should therefore be considered. Further research addressing this question, as well as the potential ability of larvae to chemically biodegrade the plastic as seen with other insect larvae (Bombelli et al., 2017), and the resulting implications, would be highly valuable.

### Implications of caddisfly behavior for the fate of plastic litter

The significant link between the amount of meso‐PLA recovered and the number of microplastics found confirms that *Agrypnia* sp. larvae can facilitate the breakdown of mesoplastic into micro‐sized particles and are therefore likely to be a previously unrecognized pathway by which microplastics are generated in freshwater systems. As noted by Mateos‐Cárdenas et al. (2020), many studies report a relatively slow breakdown of plastics from environmental weathering. For example, minimal degradation of polyethylene film was seen after 25 weeks in static freshwater (Julienne et al., 2019). Song et al. (2017) found that plastics could take up to 4.2 years to break down in a simulated beach environment, and in salt marshes the release of microplastics from larger plastic pieces only began after 8 weeks (Weinstein et al., 2016). By comparison, in our study caddisfly facilitated the rapid breakdown of plastic films over just 6 days; each larva, which had access to replete natural building material and a high‐quality food source, formed an average of 22.3 microplastics/day. Interestingly though, the degree of fragmentation and microplastic formation in our study varied considerably among individual organisms. Given that there was no relationship between larvae size and number of microplastics formed, reasons for the variability remain unclear; however, large variation in the case‐building behavior between individuals and even within the same individual is common for caddisflies (Hansell, 1968), and therefore this variability is to be expected.

Shredding organisms generally account for approximately 20% of macroinvertebrate biomass in temperate streams and rivers and play a crucial role in the degradation of large organic matter into smaller particles (Cummins et al., 1989). A range of freshwater invertebrates are known to associate with plastic litter in rivers (Wilson et al., 2021), and in the present study, preliminary evidence from outside the main experiment indicated that other caddisfly species, provisionally identified as *Limnephilus* sp., also incorporate plastic into their cases and show the same plastic fragmentation behavior as *Agrypnia* sp. (Supporting Information, Figure S6). Many other organisms therefore have the potential to contribute to plastic breakdown and microplastic release in freshwater systems, but have so far been largely overlooked, with only one other study to date having assessed whether a freshwater shredding invertebrate may use its feeding appendages to fragment plastic (Valentine & Boxall, 2022). Many studies work to develop models to map the sources, behavior, and fate of plastic in the environment (Kawecki & Nowack, 2019; Waldschläger et al., 2020), and fragmentation is already considered an important component in models such as the open source “Full Multi” modeling framework for the transport and fate of nano‐microplastics in the environment (Domercq et al., 2022). Active biological fragmentation, such as that demonstrated in the present study for caddisfly larvae, is likely to be an important consideration for the next generation of models that map the fate and transformation of plastic litter in the environment (Harrison et al., 2021). Given the findings of the present study, and the known internal fragmentation of plastic by freshwater amphipods and dragonfly larvae (Immerschitt & Martens, 2020; Mateos‐Cárdenas et al., 2020), the role of biofragmentation in macroplastic breakdown and microplastic release in freshwater systems should now receive greater research attention and be more widely considered by studies that model the dynamics of plastic in the environment. Further work with other polymer types, sizes, and concentrations is now needed to assess the potential for biofragmentation in other freshwater taxa and the extent to which it may be occurring throughout environmental systems. Mesocosm and in situ field‐based studies would be particularly useful for identifying key organisms that are most important for plastic fragmentation in freshwater, and for establishing an accurate estimation for plastic litter breakdown rate through biofragmentation pathways.

The most common size of microplastic formed by *Agrypnia* sp. larvae was between 100 and 300 µm. This is similar to the microplastics formed by the amphipod *O. gammaerellus*, of which the majority were 200–600 µm (Hodgson et al., 2018), but smaller than the particles generally formed by the sea urchin *P. lividus* (Porter et al., 2019). Although analytical methods do influence conclusions about the size of microplastics formed, the size and morphology of an organism's feeding appendages are likely to be closely linked to the size of the microplastics that are formed. Like other insects, caddisfly larvae have a well‐developed mouth, consisting of sclerotized paired mandibles, paired maxillae, and associated maxillary palps and galea, all of which function together to manipulate, guide, and fragment material (Baptista et al., 2006; Friedrich et al., 2015; Holzenthal et al., 2015). *Agrypnia* sp. larvae in the present study had serrated mandibles approximately 600 µm in length, of which the serrated portion was approximately 200 µm wide (Supporting Information, Figure S6). The resting width between the maxillary palps was approximately 200 µm, and the feeding appendages of the larvae therefore appear to be in keeping with the sizes of microplastic fragments observed. The irregular shape and notched edges of many of the microplastic fragments (Figure 3) matched the serrated area of the larvae mandible very closely, indicating that plastic was likely cut on this part. During future work to explore the microplastics formed from biofragmentation, the ability to link the size of an organism's feeding appendages with the size and shape of formed microplastics would be a valuable tool for further developing our understanding of the types of secondary microplastics being formed in the environment and the ecological risks they may pose.

## CONCLUSIONS

To the authors' knowledge, this is the first documented evidence of a freshwater invertebrate using external feeding appendages to actively fragment and physically alter plastic, therefore revealing a previously unidentified mechanism of plastic litter breakdown and microplastic formation in freshwater systems. Although the question was not directly addressed in our study, it can be concluded that interactions between caddisfly larvae and plastic films could be either beneficial or detrimental to larvae fitness, and the impacts of the resulting microplastic influx on the wider benthic community also remain unclear. Taken together, these findings suggest that to advance our knowledge of the sources, behavior, fate, and impacts of plastic pollution in freshwater systems, further research is needed to understand the extent of plastic biofragmentation behavior throughout freshwater taxa, and their relative contribution to secondary microplastic creation. Future studies should also address the choices and interactions of caddisfly larvae when presented with natural and plastic material in different stages of microbial colonization, as well as the tendency of caddisfly to utilize and fragment different plastic types present in the environment.

## Supporting Information

The Supporting Information is available on the Wiley Online Library at https://doi.org/10.1002/etc.5496.

## Conflict of Interest

The authors declare no conflict of interest.

## Author Contributions Statement

**Katey Valentine**: Conceptualization; Data curation; Formal analysis; Investigation; Methodology; Project administration; Visualization; Writing—original draft; Writing—review & editing. **Alistair B. A. Boxall**: Conceptualization; Formal analysis; Investigation; Methodology; Supervision; Visualization; Writing—original draft; Writing—review & editing. **Richard Cross**: Formal analysis; Methodology; Resources; Writing—original draft; Writing—review & editing. **Ruairidh Cox**: Data curation; Methodology; Resources. **Gina Woodmancy**: Data curation.

## Supporting information

This article contains online‐only Supporting Information.

Supporting file.

## Data Availability

Data and analysis code can be accessed in the repository: https://doi.org/10.6084/m9.figshare.20209145.
