# Supplementary material for: Caddisfly Larvae are a Driver of Plastic Litter Breakdown and Microplastic Formation in Freshwater Environments
Source: Environ Toxicol Chem. 2022 Nov 18;41(12):3058–69. doi: 10.1002/etc.5496 (PMC9827824; doi:10.1002/etc.5496)
Supplement: Supplementary file 1 — Supporting file. [file ETC-41-3058-s001.docx]

**Supplementary material**

**Caddisfly larvae are a driver of plastic litter breakdown and microplastic formation in freshwater environments**

Katey Valentine *et al.*

Corresponding author: [klv501@york.ac.uk](mailto:klv501@york.ac.uk)


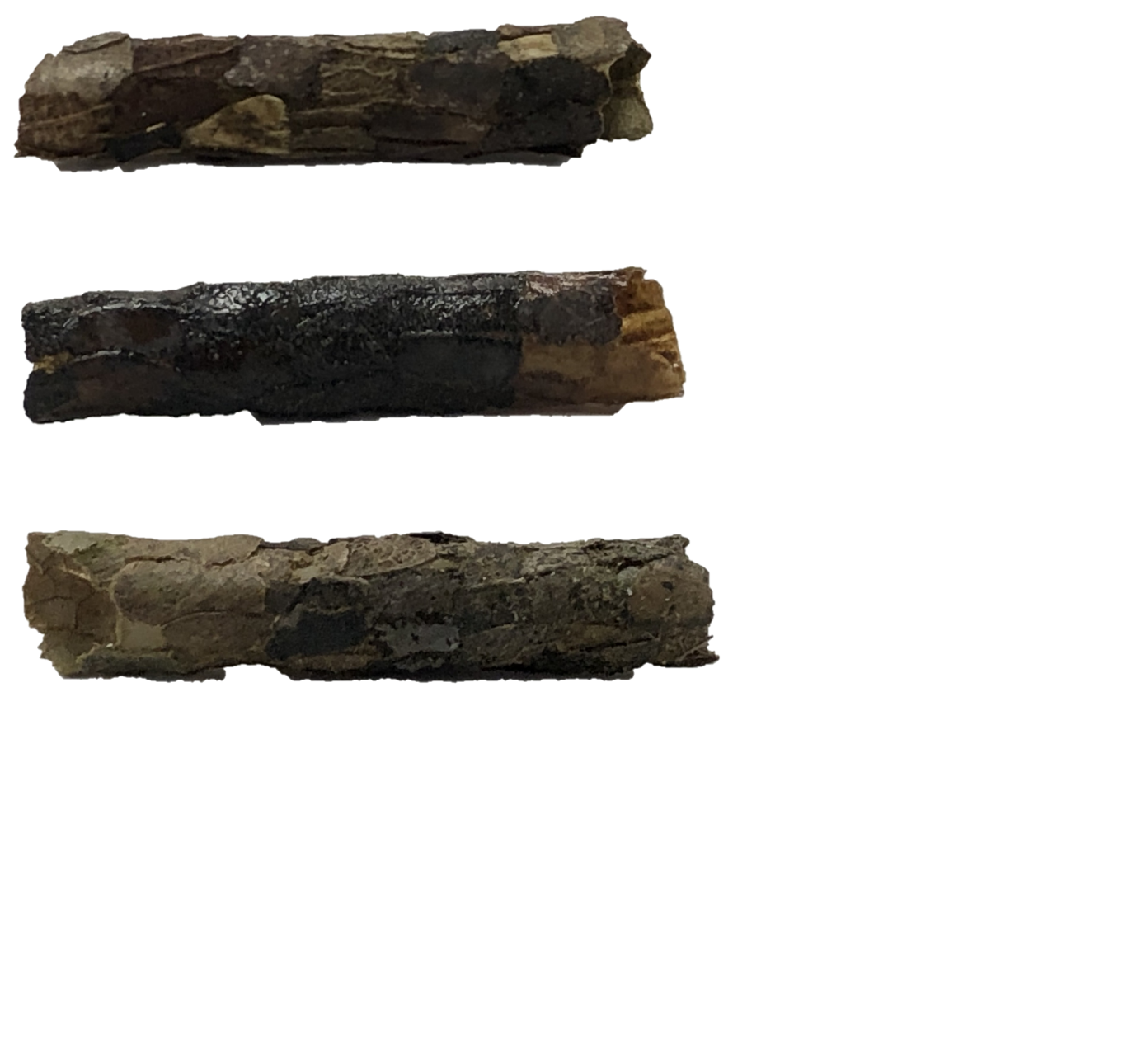
**Supplementary figure S1**. Examples of the original cases that *Agrypnia* sp. were removed from - built from fragmented flexible organic material.

**Supplementary methods S1.**

Modified algal pellet recipe from (Kampfraath et al., 2012):

Algal pellets were made by boiling 12g of nutrient agar with 500ml deionized water before adding 15g of cellulose and 15g of food-grade *Chlorella* powder and 5mg ascorbic acid. The pellet mixture was homogenised, poured into a glass dish, covered and then cooled for 30 minutes before being cut into uniform 0.95g cylindrical pellets.

**Supplementary methods S2**

To confirm the visual counting correctly identified PLA fragments, micro-Fourier transform infrared spectroscopy (μFTIR) was used to confirm the polymer identity of the white fragments. Both control samples (containing no added PLA) and samples from the exposure waters were analysed. Samples were thoroughly mixed by vigorously shaking for 10 s, then immediately deposited onto a 25 mm diameter 3 μm pore size silver membrane filter (Sterlitech, Washington USA) using a glass pipette. Where the entire sample could not be deposited, a sub-sample was determined by weight to 0.1 mg accuracy.

All particles within the deposition area (about 11 x 11 mm) were identified and quantified with an imaging μFTIR spectrometer (PerkinElmer Spotlight 400) set to collect spectra in the range between 4000 and 700 cm−1 wave numbers. A background spectrum of the silver filter was collected prior to each sample and removed from resulting data. A first analysis scanned the whole area at a pixel size of 25 µm to give a reasonable compromise between resolution, processing time and resulting file size. The minimum particle size that can be enumerated in these scans is therefore 25 µm. A higher resolution scan on selected areas of the filter was then also performed at 6.25 µm pixel size (the lowest resolution pixel size on the Spotlight FTIR system, and near the theoretical spatial limits of conventional FTIR imaging), enabling the imaging and enumeration of particles smaller than 25 µm. All mapping was carried out at a resolution of 8 cm−1, with two scans per pixel, and an interferometer speed of 2.2 cm/s.


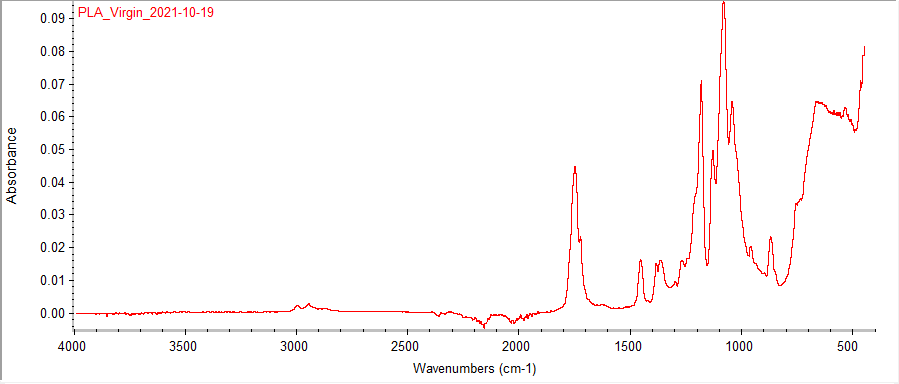
The generated spectra were analysed using the siMPle software (http://simple-plastics.eu) Spectra were matched against an expanded polymer database, as developed and described by (Primpke et al., 2018). Spectra were matched against the database at an initial Pearson’s correlation coefficient value of 0.65 (where 1.0 is a perfect match and 0 is a complete mismatch) against the raw and first derivative spectra, with subsequent particle building thresholds of 0.4 and 0.3. This means that a lower level of certainty is accepted for a pixel that is adjacent to an already positively identified pixel of the same polymer.

**Supplementary figure S2.** FTIR spectra of the polylactic acid film used throughout the study.

**Supplementary table S1.** Details of all statistical tests carried out in the study and any transformation that were made to meet assumption of normality and equal variance.

| **Comparison** | **Transformation** | **Test** |
| --- | --- | --- |
| Old case length | None | Unpaired, two-tailed t-test (equal variance) |
| New case length | None (normality not achieved through transformation) | Mann-Whitney U |
| Surface area of total material used to build new case | None | Unpaired, two-tailed t-test (equal variance) |
| Wet weight of larvae at end | None | Unpaired, two-tailed t-test (equal variance) |
| Amount of plastic used compared to leaf within each treatment | None | Paired, two-tailed t-test (equal variance) |
| Amount of PLA used ML vs MR | None | Unpaired, two-tailed t-test (equal variance) |
| Amount of leaf used ML vs MR | None | Unpaired, two-tailed t-test (unequal variance) |
| Proportion of meso-PLA pieces that were intact between ML and MR | None | Unpaired, two-tailed t-test (equal variance) |
| Size of intact, ML chewed and MR chewed pieces | None (normality not achieved through transformation) | Kruskall Wallis, followed by a Dunn’s post hoc test |
| Number of microplastics found between ML and MR | Square-root | Unpaired, two-tailed t-test (equal variance) |
| Regression analysis between number of PLA microparticles and surface area of meso PLA recovered ML treatment | None | Linear regression |
| Regression analysis between number of PLA microparticles and surface area of meso PLA recovered MR treatment | None | Linear regression |
| Regression analysis between number of PLA microparticles and larvae weight ML treatment | None | Linear regression |
| Regression analysis between number of PLA microparticles and larvae weight ML treatment | None | Linear regression |
| Difference between micro-particle size between treatments | None (normality not achieved through transformation) | Mann-Whitney U |

**Supplementary video 1.**

DOI: 10.6084/m9.figshare.21080662

Video of a *Agrypnia* sp. larva chewing and fragmenting polylactic acid film, before incorporating it into its protective case. Video was recorded for a larva outside of the main study, during preliminary trials. Video is edited together clips from a longer video which records this behaviour over a period of around 45 minutes and is shown at a playback speed of times 3.


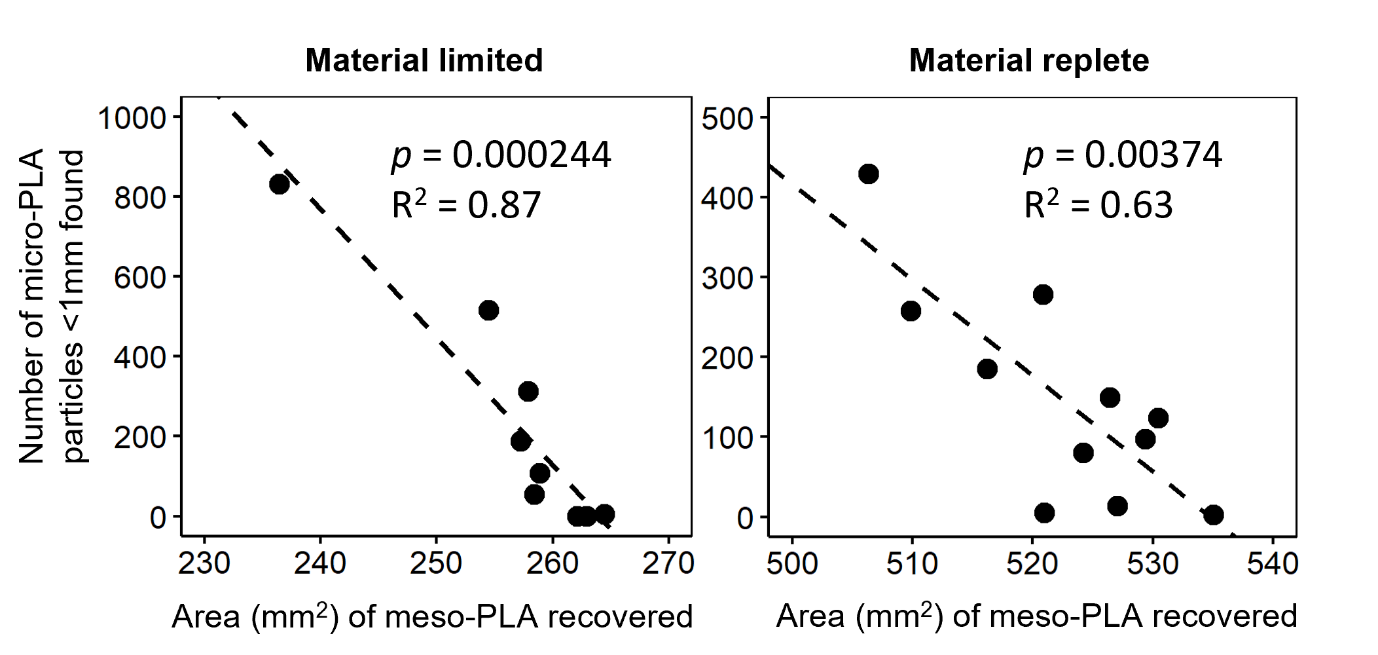


**Supplementary figure S3**. Number of micro-PLA particles found in the exposure water plotted against the total surface area of meso-PLA pieces recovered from the case and exposure jar for each replicate of material limited (*n* = 9) and material replete (*n* = 11) treatments. Significance (*p*) value of the regression test and R^2^ values are given on each plot.


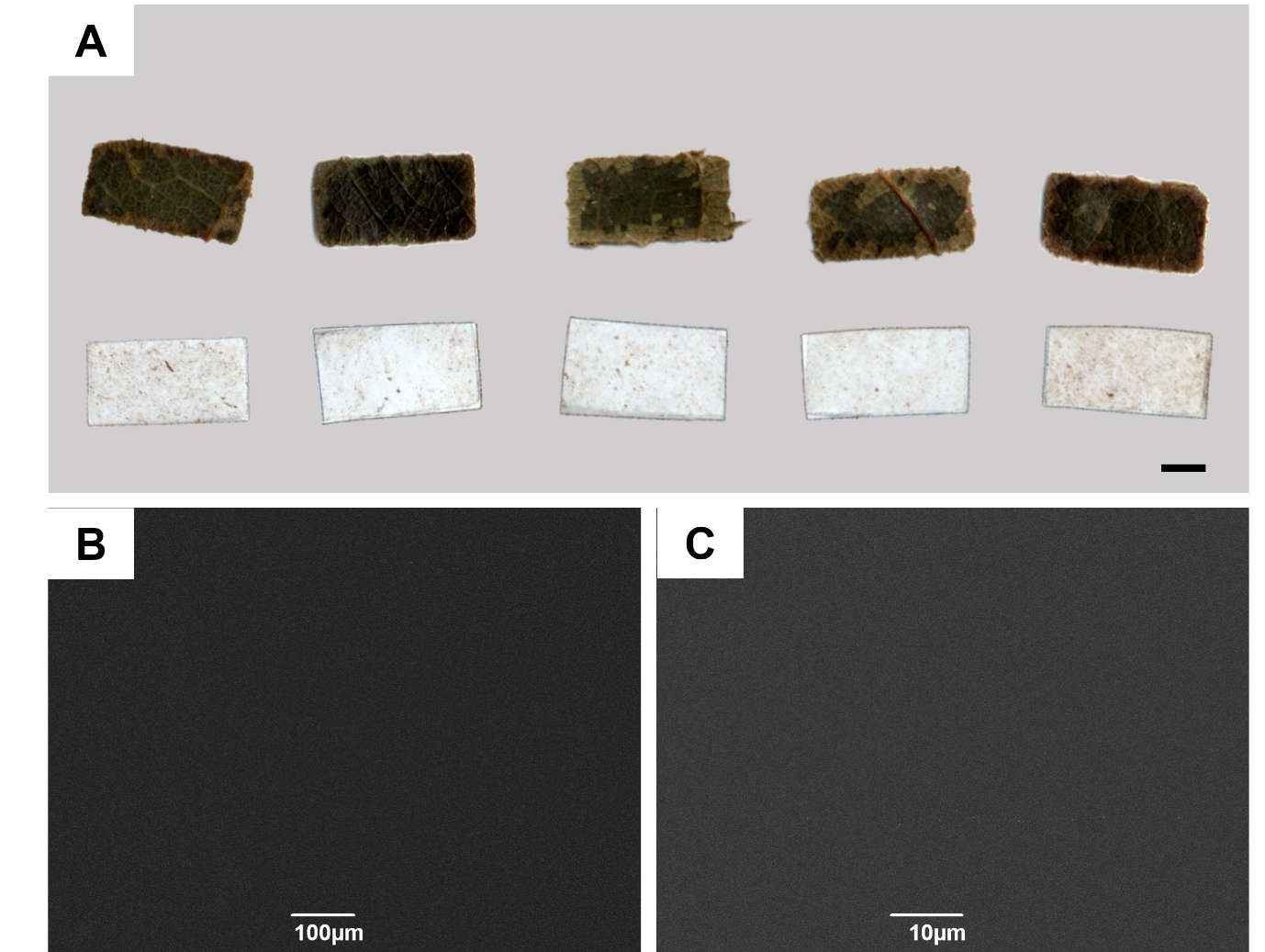
**Supplementary figure S4.** Images of polylactic acid film (PLA) and *Quercus robur* surfaces. (A) Coupons of PLA and *Q. robus* provided to caddisfly – note the textured surface of the *Q. robus* leaf material, scale bar = 10mm. (B) & (C) Scanning electron microscope (SEM) images of the surface of virgin PLA. To be directly compared with the SEM image of the surface of mature *Q. robus* leaves presented in (Gülz & Boor, 1992) - figure 5.


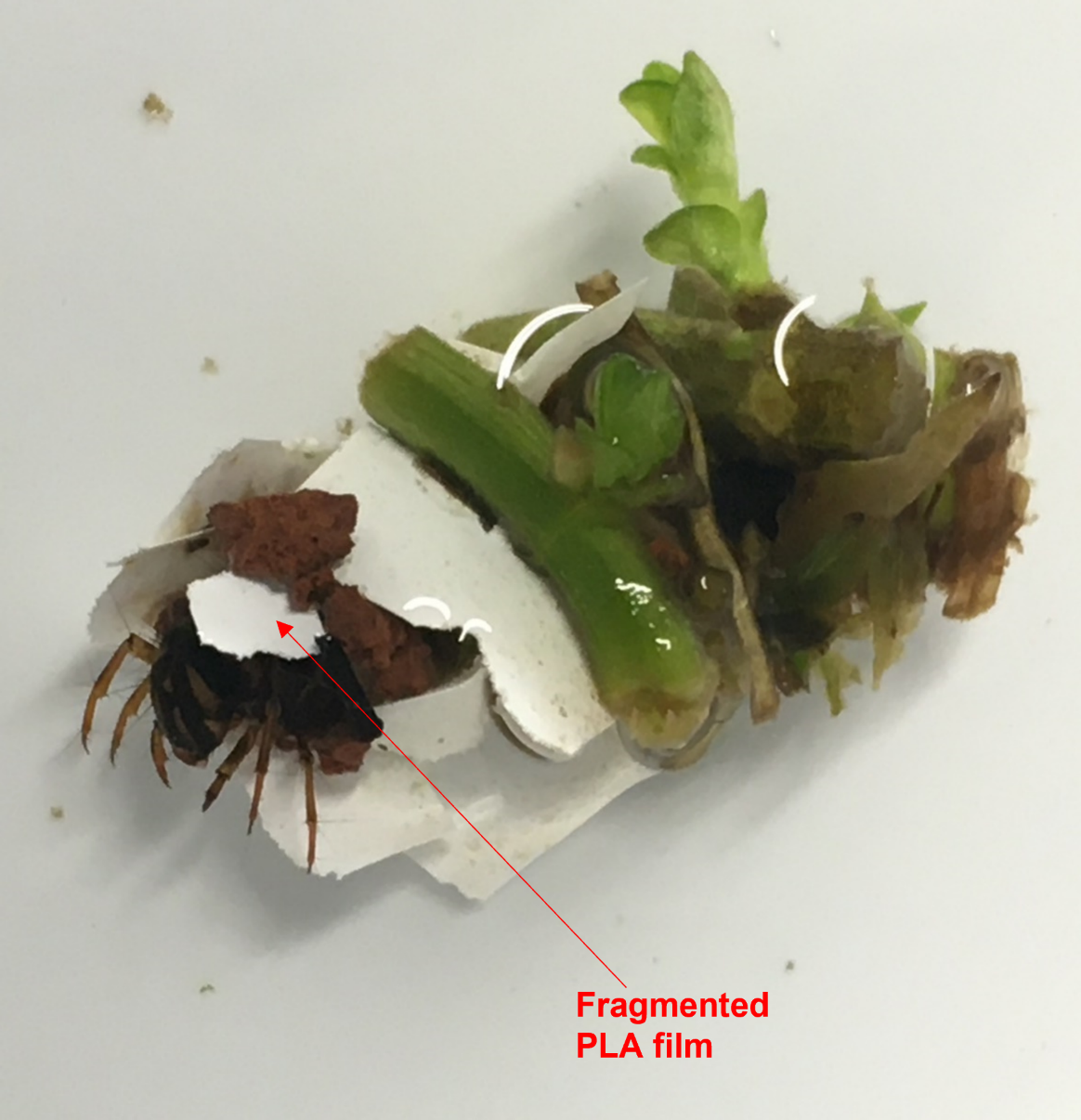


**Supplementary figure S5.** Preliminary evidence of another caddisfly species, provisionally identified as *Limnephilus* sp., fragmenting and using PLA film in their case.


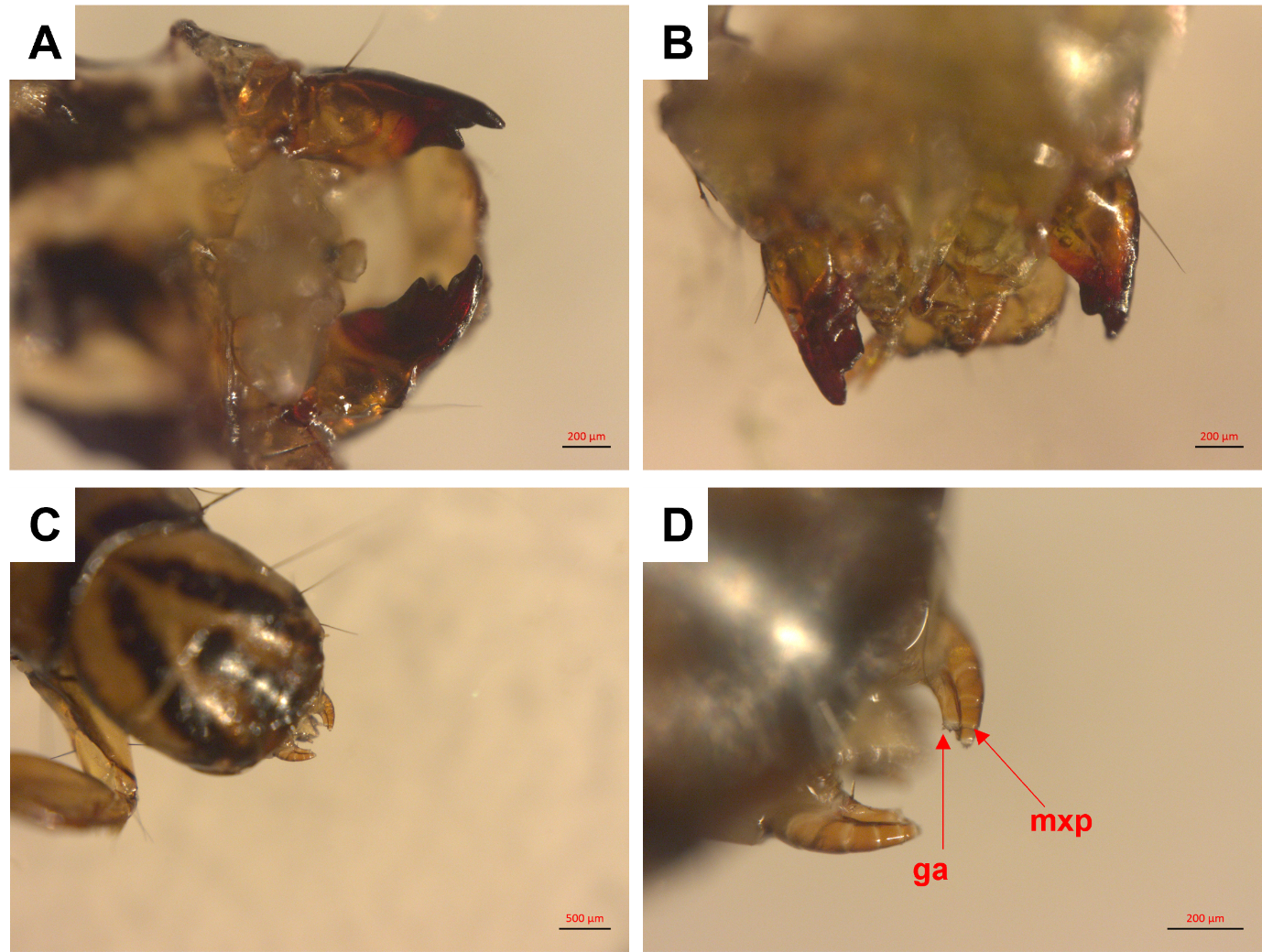


**Supplementary figure S6.** Feeding appendages of *Agrypnia* sp. (A) & (B) show the serrated mandibles used to cut material. (C) & (D) show the maxillary palps (mxp) and galea (ga) which work in conjunction with the mandibles to sense and guide material.

**References**

Gülz, P.-G., & Boor, G. (1992). Seasonal Variations In Epicuticular Wax Ultrastructures Of Quercus Robur Leaves. *Zeitschrift Für Naturforschung C*, *47*(11–12), 807–814. https://doi.org/10.1515/znc-1992-11-1205

Kampfraath, A. A., Hunting, E. R., Mulder, C., Breure, A. M., Gessner, M. O., Kraak, M. H. S., & Admiraal, W. (2012). DECOTAB: A multipurpose standard substrate to assess effects of litter quality on microbial decomposition and invertebrate consumption. *Freshwater Science*, *31*(4), 1156–1162. https://doi.org/10.1899/12-075.1

Primpke, S., Wirth, M., Lorenz, C., & Gerdts, G. (2018). Reference database design for the automated analysis of microplastic samples based on Fourier transform infrared (FTIR) spectroscopy. *Analytical and Bioanalytical Chemistry*, *410*(21), 5131–5141. https://doi.org/10.1007/s00216-018-1156-x
